# Subjective Poverty Moderates the Association Between Carer Status and Psychological Outcomes of Adult Siblings of People with Intellectual and Developmental Disabilities

**DOI:** 10.1007/s10803-022-05520-3

**Published:** 2022-03-19

**Authors:** Nikita K. Hayden, Richard P. Hastings, Clare Kassa, Frances Danylec

**Affiliations:** 1 Centre for Educational Development, Appraisal and Research, University of Warwick, New Education Building, Coventry, CV4 7AL UK; 2 Department of Psychiatry, Centre for Developmental Psychiatry and Psychology, Monash University, Melbourne, Australia; 3 Sibs, UK-wide charity, Keighley, UK

**Keywords:** Adult siblings, Psychological outcomes, Health outcomes, Caregiving, Intellectual disabilities, Developmental disabilities

## Abstract

Adult siblings are potentially important sources of care, support, advocacy, and friendship for their brothers and sisters with intellectual and developmental disabilities (IDD). Drawing on data about 851 adult siblings who completed an online national survey, we examined predictors and potential key moderators of siblings’ mental distress, wellbeing, quality of life, and health outcomes. Moderated regression analyses indicated that siblings experiencing higher levels of subjective poverty; siblings with brothers and sisters with lower levels of independence; and siblings who are carers and also experiencing low levels of subjective poverty, had worse outcomes and may be in need of specific supports. Compared to normative samples, adult siblings of people with IDD had worse outcomes across the measures.

## Introduction

People with intellectual and developmental disabilities (IDD) are living longer lives than ever before (Coppus, 2013), with many people with IDD expected to outlive their parents’ ability to provide care. Future planning for people with IDD often considers siblings as potential providers of care and support for their brothers and sisters with IDD (Lee & Burke, 2020). Understanding the psychological and health outcomes of adult siblings is important because siblings may provide care, support and friendship for their brothers and sisters with IDD (Lee & Burke, 2018), and will be more prepared for these roles if they are psychologically well and physically healthy. There is also a question about whether adult siblings may be at risk of worse outcomes themselves compared to other adults. This question would be consistent with family systems perspectives, whereby family members are understood to influence and impact one another (Cox & Paley, 1997).

With the increased life expectancy amongst people with IDD, and the likelihood that some siblings will take on caring later in life when their parents are no longer able to (Lee et al., 2019), it is foreseeable that siblings may become more involved with the care of their brothers and sisters with IDD as they are facing more age-related health problems such as physical health and mobility needs, behavior changes, and dementia (Coyle et al., 2014). Sibling caregiving may also be unique, because siblings may anticipate and mentally prepare themselves for taking on a caregiving role from a young age. Furthermore, siblings may be more likely to be ‘compound’ carers (Lee et al., 2020), whereby they may care for their own children, their own parents, and their brothers and sisters with IDD, therefore managing multiple, distinct caregiving roles. Although positive aspects related to caregiving must be highlighted (Heller & Kramer, 2009; Lee & Burke, 2018), it is important to more fully understand the outcomes of sibling caregivers using validated measures to help us further understand whether sibling carers have support needs and what those might be.

Many existing studies about the outcomes of adult siblings have relied on small, convenience-based samples without a normative comparison group, making it difficult to fully contextualize findings (e.g., Howlin et al., 2015; Tomeny et al., 2017; and O’Neil & Murray, 2016). Some larger-scale studies have been published. For example, Rai et al. (2018), drawing on a Swedish population-based cohort study of 223,842 participants, compared depression in young adults with and without autism. Part of their analyses drew on depression diagnoses in the cohort members’ full and half siblings. They found that the full and half siblings of young adults with autism were at a slightly increased risk compared to the general population of having a depression diagnosis even when other socio-demographic factors (e.g., sex, age, family income) were considered (adjusted risk ratio for full-siblings = 1.37; adjusted risk ratio for half-siblings = 1.42). Taylor et al. (2008) drew on data from the Wisconsin Longitudinal Study to compare the outcomes of 268 adult siblings of people with mild ID, 83 adult siblings of people with mental illness, and 791 siblings of people without a disability. Taylor et al. found that adult siblings of people with mild intellectual disability had similar outcomes to adult siblings of people without disabilities. These inconsistencies in findings relating to increased risk of negative outcomes for adult siblings may be associated with the geographical, generational, and disability-type differences in these two studies.

A literature review by Heller & Arnold (2010) further emphasized inconsistencies in research findings about adult siblings. In terms of the psychosocial outcomes of adult siblings, results were mixed, with eight studies highlighting positive psychosocial outcomes, and five studies showing more negative psychosocial outcomes for siblings. These differences in findings emphasize the need for analyses that consider questions about under what conditions some siblings experience worse or better outcomes. Such information might better inform appropriate support for adult siblings. This requires moving beyond analyses that explore direct effects of different risk factors to also consider moderated effects. We found two studies that used moderation analyses to explore the outcomes of siblings. Prino et al. (2019) found that gender moderated the association between sibling relationship quality and coping strategies. O’Neill & Murray (2016) found that none of their interaction terms moderated the association between being a sibling of someone with IDD and sibling depression or anxiety outcomes. Their moderation variables included: gender, socio-economic status (SES), age, and education levels.

Adult siblings may have differing outcomes based on whether they are a carer for their brother or sister with IDD. Much research about adult siblings as carers has concentrated on predicting caregiving (Burke et al., 2012), future planning (Lee & Burke, 2020), and identifying support needs (Arnold et al. 2012). Less is known empirically about the outcomes of adult sibling carers as a specific sub-group of adult siblings, although Arnold et al. (2012) have speculated that the more involved in care siblings are, the more support needs siblings may have themselves. A systematic review by Lee & Burke (2018) identified sibling caregiving challenges, such as those related to emotional, economic, and physical needs; family conflict; and worries about challenges arising from ageing; as well as navigating services. One of the studies in the systematic review (Taggart et al., 2012), identified that over half of their sample (*N* = 112) of parent and sibling carers of people with IDD reported that caring was physically as well as emotionally demanding, with 40% self-reporting anxiety, and 31% self-reporting depression. A population-representative study about informal carers of people with intellectual disabilities (ID) explored the health, quality of life and impact of caring on carers of people with ID in comparison to other carers (Totsika et al., 2016). Although the types of relationships between the people with ID and their ‘informal carers’ were not reported, many of these carers would have been family members, including possibly adult sibling carers. Totsika et al. (2016) found that carers of people with ID had a similar quality of life to other carers, although they were at an increased risk of poorer health and were more likely to be struggling financially, which was related to poorer quality of life, health status, impact on health, and impact on personal life.

It is unclear in the sibling research literature what the psychological and health outcomes are for sibling carers specifically, and what is associated with the outcomes of sibling carers. Therefore, further research is needed to understand not just adult siblings’ psychological and health outcomes, but also the outcomes of sibling carers. In addition, it is important to consider what factors are associated with carer and non-carer adult siblings’ outcomes, and how these factors interact.

Few studies identified in Lee & Burke’s (2018) systematic review about caregiving roles of adult siblings included measures of socio-economic status (SES) and financial hardship (three of 29 studies included income). Of the three, Sonik et al. (2016) included and went beyond income to explore various aspects of socio-economic status, including poverty, employment status, food security, receipt of benefits, and material hardship. Sonik et al. (2016) provided a descriptive comparison about adult sibling carers that lived with their brother or sister with IDD as the head of the household or partner of the head of the household (*n* = 78) and compared them to working-aged adults (*n* = 64,555) using USA, population-level, multi-wave data. They found that adult sibling carers were more likely to experience poverty and socio-economic hardships. Statistically significant comparisons found that sibling carers were more likely to be living 300% below the USA Federal poverty level, to not be employed in the past 5 weeks, to experience low or very low food security, and to receive public benefits compared to other adults of working age. Sonik et al. (2016) also explored education level and found that sibling carers were more likely to be less educated. Little work has been done in the caregiving or adult sibling literature about the association between SES and mental wellbeing and physical health outcomes, although the child sibling literature indicates that SES is an important covariate in understanding siblings’ psychological outcomes (Hayden et al., 2019). Measuring SES presents a methodological challenge and moving beyond income and using cumulative measures of SES is an important way of capturing variations and nuances in SES amongst populations (Galobardes et al., 2007) and few sibling studies have explored this concept extensively. Further work is needed to not only incorporate a range of SES measures in sibling studies, but also to understand what role SES plays in the association between caregiving and sibling outcomes beyond exploring simple direct effects.

Another important factor that needs to be understood in relation to adult sibling outcomes and caregiving is the level of independence skills that the brother or sister with IDD has. Studies have explored whether the level of independence is associated with whether the sibling is already, or anticipates becoming, a caregiver for their brother or sister with IDD. These findings were mixed according to Lee & Burke’s (2018) systematic review: they identified two studies finding that siblings were more likely to anticipate caring for their brothers and sisters with IDD when they had higher levels of independence (Rimmerman & Raif, 2001), one study that found they were more likely to anticipate caring if their brothers and sisters with IDD had lower levels of independence (Seltzer et al., 1991), and one study found no statistically significant difference based on this factor (Heller & Kramer, 2009). Less considered in the existing sibling literature, is the level of independence of the brother or sister with IDD and what role sibling independence skills play in the relationships between caregiving and the mental wellbeing and physical health outcomes of adult siblings. The non-sibling caregiving literature indicates that the more severe the disability of the person they are caring for, the worse outcomes caregivers will experience (e.g., parental caregivers of people with IDD, Seltzer et al., 2010). Those with lower levels of independence are likely to need more support and care.

Overall, there is a lack of existing evidence about the outcomes of siblings that are carers and non-carers, and that provide meaningful comparisons between adult sibling carers, adult sibling non-carers, and other adults. What other factors may be associated with adult sibling carer and adult sibling non-carer outcomes have also rarely been studied. The existing literature provides some support for considering how factors such as SES and the level of independence of the brother or sister with IDD may moderate the association between whether the sibling is a carer, and their psychological and health outcomes.

Therefore, we sought to explore the following research questions: (1) Does SES and the level of independence of the brother or sister with IDD moderate the association between sibling carer status and siblings’ mental distress, wellbeing, quality of life, and health outcomes? (2) What are the mental distress, wellbeing, quality of life, and health outcomes of adult siblings, and how do these compare to other adults? For the first research question, drawing on the Double ABCX model (McCubbin & Patterson, 1983), we expected to find that both SES and the level of independence would moderate the association between siblings being a carer and their mental distress, wellbeing, quality of life, and health outcomes. Regarding the second research question, although findings in existing research vary, we expected to find that siblings may have slightly worse psychological scores (i.e., mental distress, wellbeing) compared to other adults (Rai et al., 2018). For quality of life, we were only able to compare our sibling sample to other informal carers, so we expected to find similar levels of quality of life to other carers (Totsika et al., 2016). For health, Hodapp et al., (2010) found that most adult siblings reported good health, but we do not yet know how adult siblings’ health compares to other adults.

## Method

### Participants

The sample consisted of 851 adult siblings of people with IDD. The mean age of adult siblings was 34.75 years (*SD* = 12.76, Range = 18–76 years). To take part, participants had to be aged 18 years and over, had to live in the UK, have a brother or sister with IDD, and had to consent to take part in the research. Adult siblings were predominately female (84.7% female; 14.6% male; 0.6% a gender not listed; 0.1% missing). Data from the UK Indices of Multiple Deprivation (IMD) measure relative deprivation of small geographic areas by utilizing national Census data (Gill, 2015). These data are available publicly for each country in the UK whereby entering participant postcodes (i.e., zip codes) produces a decile for the local area indicating whether participants live in the least to the most deprived neighborhoods in their respective countries. These deprivation data draw on seven different domains of deprivation and includes measures of income, employment, education, health, crime, housing, and environment. For the adult siblings in this sample, 10.9% were living in the 20% (bottom two deciles) most deprived neighborhoods in the UK (13.7% missing). In terms of health, 30.2% of adult siblings indicated that they had a longstanding illness, disability, or infirmity. The largest ethnic group in the sample was White British (*n* = 773, 90.8%), White Irish (*n* = 17, 2.0%) and White Other (*n* = 17, 2.0%). The next largest groups were Asian/Asian British Pakistani (*n* = 5, 0.6%), Asian/Asian British Indian (*n* = 5, 0.6%), Mixed/multiple ethnic group: White and Black Caribbean (*n* = 5, 0.6%), and Mixed/multiple ethnic groups: White and Asian (*n* = 5, 0.6%). The largest religion group were ‘No religion’ (55.6%), followed by Christian (all denominations; 38.1%). The majority of the sample lived in England (87.5%), with 5.8% living in Scotland, 4.3% living in Wales, and 1.5% living in Northern Ireland (0.8% missing).

Adult siblings also provided background information about their brother or sister with IDD. The mean age of their brothers/sisters with IDD was 34.01 years (*SD* = 12.87, Range = 16–83 years), 48.2.0% were male, 32.7% were female; 0.8% identified as a gender not listed, and data were missing for 18.3%. Brothers/sisters with IDD were most commonly identified (non-exclusive categories) as having intellectual disability (49.0%), Autism (38.3%), Down syndrome (29.0%), or Cerebral Palsy (7.8%). Adult siblings were also asked to identify if their brothers/sisters with IDD had problems with various areas of physical health: 25.3% had visual impairments, 16.8% had hearing impairments, 34.3% had mobility problems, 40.7% had physical health problems, and 21.5% also had ‘epilepsy/seizures’.

### Recruitment

Participants were recruited from October 2019 until February 2020.  Sibs,  a UK-wide non-profit organization for brothers and sisters of disabled children and adults, led recruitment, advertising through their mailing list and through their social media networks (i.e., Twitter and Facebook).  Sibs also contacted relevant IDD and family support organizations to encourage dissemination amongst further mailing lists and social media networks beyond Sibs’ network.

Initially, there were 927 participants who responded to the survey, but 13 cases were removed as they had consented but then not responded to any of the survey questions. A further three participants were removed because they were residing outside of the UK and our ethical approval only allowed us to recruit UK participants. A further 60 participants were removed from this analysis because their brother or sister with IDD was aged under 16 years of age, resulting in an overall sample of 851 siblings. Those whose brother or sister was under 16 years of age were removed from the sample to reduce the chance that adult siblings identifying themselves as carers would not have had in mind ordinary supports relevant to children (e.g., regular babysitting). All participants completed the survey online. There was no financial incentive offered to participants.

### Measures

#### Adult Sibling Outcomes

Adult sibling mental distress was measured using the Kessler 6 (K6; Kessler et al., 2003). Participants were asked how often they have felt in the last 30 days: ‘nervous’, ‘hopeless’, ‘restless’, ‘fidgety’, ‘so depressed that nothing could cheer you up’ and whether everything was an ‘effort and worthless’. Participants respond on a five-point scale ranging from ‘all of the time’ to ‘none of the time’. These items are summed with scores ranging from zero to 24 and higher scores indicated more mental distress. We allowed one missing item to be replaced with mean estimation. Internal consistency (Cronbach’s α) for the K6 was 0.88 in the current sample.

Adult sibling wellbeing was measured using the Short-form Warwick Edinburgh Well-Being Scale (SWEMWBS; Stewart-Brown et al., 2009). This consists of seven, positively phrased items such as ‘I’ve been feeling optimistic about the future’ and ‘I’ve been feeling close to other people’. Participants were asked to rate these seven items on the following five-point scale based on their experiences in the preceding 2 weeks: ‘None of the time’, ‘Rarely’, ‘Some of the time’, ‘Often’, or ‘All of the time’. If there were any missing data for a participant, a raw score was not produced. Raw scores were then transformed using SWEMWBS guidance to facilitate cross-study comparisons, and to benefit from superior scaling properties (Stewart-Brown et al., 2009). Final scores for the SWEMWBS were on a scale of seven to 35, with higher scores indicating better adult sibling wellbeing. Internal consistency (Cronbach’s α) for the SWEMWBS was 0.85.

Adult siblings’ quality of life was measured using a five-point scale of ‘Very good’, ‘Fairly good’, ‘Neither good nor bad’, ‘Fairly bad’ or ‘Bad’ in response to the following question: ‘If we were to define ‘quality of life’ as how you feel overall about your life, including your standard of living, your surroundings, friendships and how you feel day-to-day, how would you rate your quality of life?’ This question has been used in major UK national surveys such as the ‘*Survey of Carers in Households, *2009–2010*’* (Information Centre for Health and Social Care, GfK NOP, 2011).

Adult sibling health was measured by asking siblings to self-assess their ‘health in general’ on a five-point scale: ‘Very good’, ‘Good’, ‘Fair’, ‘Bad’ or ‘Very bad’ with higher scores indicating worse perceived health. This question has also been used in major national UK surveys including the ‘Survey of Carers in Households, 2009–2010’ (Information Centre for Health and Social Care, GfK NOP, 2011) and the ‘Health Survey for England, 2018’ (2020).

#### Main Predictor Variables

Adult sibling participants were asked to identify (Yes or No) whether they were a carer for their brother/sister with IDD based on the following definition used nationally in the UK: ‘A carer is anyone, including children and adults, who looks after a family member, partner or friend who needs help because of their illness, frailty, disability, a mental health problem or an addiction and cannot cope without their support. The care they give is unpaid’ (NHS England, 2020).

Subjective poverty was measured by incorporating responses from two questions. Participants were asked how easily they could raise £2000 in an emergency and responded on a four-point scale whether they could ‘easily’ raise the funds, could do so by making ‘some sacrifices’, could do so by doing ‘something drastic’ or that they could not raise the money. This measure has been used in large-scale national surveys (Australian Institute of Family Studies, 2011). Participants were also asked to respond on a five-point scale to how well they were ‘managing financially these days’: ‘Living comfortably’, ‘Doing alright’, ‘Just about getting by’, ‘Finding it quite difficult’ or ‘Finding it very difficult’. This question was drawn from the Millennium Cohort Study (MCS, 2017), a UK nationally representative longitudinal study. This latter variable was moderately skewed. When deciding how to transform this variable, we considered the constructs and limitations of our variables measuring socio-economic status and poverty. Both of these constructs were “subjective” measures of poverty and perceived hardships. Therefore, there was a conceptual reason to combine these items into one variable. Therefore, the responses to these two items were z-transformed, then summed and divided by two (i.e., the number of items) to produce a single index of subjective poverty.

The level of independence in daily living of the brothers/sisters with IDD was measured using the Waisman Activities of Daily Living Scale (W-ADL; Maenner et al., 2013). Adult sibling respondents were asked to rate the level of independence their brother or sister with IDD had across 17 items on a three-point scale, ‘Independent or does on own’, ‘Does with help’, or ‘Does not do at all’ on statements including: ‘Making their own bed’ or ‘Doing errands, including shopping in stores’. Items were coded zero to two and summed so that total W-ADL scores ranged from zero to 34, with higher scores indicating that their brother/sister with IDD had higher levels of independence in their daily living. We made a small adjustment to the measure so that participants could also select ‘I do not know’ (ultimately coded as ‘missing’) to acknowledge that some siblings may not know about all aspects of their brother/sisters with IDD’s level of independence, particularly if they were not a carer. The original measure asks participants to answer about their son/daughter and we changed this wording to brother/sister. We allowed a total W-ADL score to be calculated in cases where up to three items were missing, using mean estimation. Internal consistency (Cronbach’s α) for the W-ADL was 0.94 in the current sample.

### Procedure

Data were drawn from an online survey using Qualtrics™. The survey was designed through collaboration with Sibs. Although three of the team that contributed to designing and scoping the survey were adult siblings, we also sought feedback from an additional four adult siblings who had not been involved in the survey design. Full ethical approval was granted by the University of Warwick’s Humanities & Social Sciences Research Ethics Committee. Online advertisements and social media postings contained a link to the study information sheet and consent form on Qualtrics™. Participants were asked to read the information sheet and to agree to each consent statement to progress onto the survey. The majority of participants that began the survey completed it until the end, with 646 of 851 participants completing the final survey item.

### Analysis Procedure

All analyses were conducted using IBM SPSS Statistics 27. In terms of missing data, 14%–15% of participants had a missing score on each key outcome used in the analysis (K6—Mental distress, 14.6%–14.8% missing; SWEMWBS—Mental wellbeing, 14.6%–15% missing; Quality of life, 15.6% missing; and Health, 14.1% missing). This missingness was related to participants dropping out of the survey during the income and finance related questions, and then not completing any of the outcome measures. We used listwise deletion of missing data because we had a relatively large sample, and it was not appropriate to model any missing data given the likely connection with participants’ lack of willingness to provide some survey data. We examined the distribution of the data which led to us dichotomizing several continuous variables that were non-normally distributed (i.e., number of disabled siblings, number of non-disabled siblings, neighborhood deprivation (IMD). Skewness and Kurtosis statistics indicated that all other variables were normally distributed. We also examined residuals from the normality plots for each regression model and these provided further support that variables had acceptable normality distributions. We also tested for multicollinearity between variables which resulted in the removal of two variables from the final moderated regression analyses. These variables were the number of health conditions of the sibling with IDD and the age of the siblings with IDD as these were highly associated with the level of independence of the person with IDD (W-ADL) and the age of the sibling respondents respectively. Collinearity statistics indicated minimal issues related to multicollinearity between the variables included in the multiple regression models (Tolerance range = 0.527–0.951; VIF range = 1.052–1.898).

To examine our first research question, whether SES and the level of independence of the brother or sister with IDD moderates the association between sibling carer status and siblings’ outcomes, we first, explored whether each of the covariates were associated with the four sibling outcomes. Any that were not significantly associated with at least one of the four adult sibling outcomes were removed from further analyses. We then examined the remaining covariates to establish if any further variables should be removed due to multicollinearity, leading us to remove the age of the brother or sister with IDD as it was highly correlated with the age of the sibling (*r* = 0.92, *N* = 694, *p* < 0.001 two tailed). The age of their brother/sister with IDD was removed because it was slightly less associated with the sibling outcomes and because, conceptually, this study focused on the outcomes of the adult sibling rather than their brother/sister with IDD.

We then used moderated multiple regression models (Hayes, 2018; PROCESS macro; version 4.0; Model 2) to examine our first research question. We used mean centered product terms for the interactions. We included a range of covariates in our analyses based on commonly explored correlates in the existing sibling and broader caregiving literature. We also considered theoretical rationales for including these covariates, driven by family systems theory. Covariates included: Age of both siblings (Heller & Kramer, 2009; Lee & Burke, 2020); whether the adult sibling participant was older or younger than their brother/sister with IDD (Egan & Walsh, 2001); the gender of both siblings (Sonik et al., 2016); whether they were the same or a different gender to one another (Heller & Kramer, 2009); whether the brother/sister with IDD had Down syndrome, autism (Hodapp & Urbano, 2007; Orsmond & Seltzer, 2007), or Cerebral Palsy; whether the adult sibling participant lived in the parental home and whether they lived with their brother/sister with IDD (Sonik et al., 2016); whether or not they had at least one non-disabled sibling in addition to their sibling with IDD (Burke et al., 2012; as they may provide support or reduce carer burden); whether they had one or more disabled siblings in addition to having a brother/sister with IDD (a broader caregiving study found that people caring for two or more people were at a higher risk of poorer quality of life and health; Totsika et al., 2016); and additional measures of socio-economic position including having a degree-level qualification and living in a deprived neighborhood (Sonik et al., 2016).

To answer our second research question regarding how the outcomes of adult siblings compare to other adults, we conducted *t* tests to summarize adult siblings’ outcomes on the following outcomes: mental distress, wellbeing, quality of life, and health. These measures have been used in existing national population surveys, so we were able to summarize and compare adult siblings’ outcomes to normative samples of adults.

## Results

### Research Question 1

#### Bivariate Analyses

Table 1 explores the associations between each predictor/covariate and the four sibling outcomes. The tests presented in this table were used to decide which variables we would include as covariates in the moderated multiple regression models, and to check whether our test variables (carer status, SES, and level of independence of the brother or sister with IDD) were associated with the outcome variables. Variables that were statistically significantly associated with at least one of the four sibling outcome variables were retained in the moderated multiple regression models for all outcomes.

**Table 1:** Associations between potential covariates and outcomes

	Mental distress		Wellbeing		Quality of life		Health	
	*t*	df	*t*	df	*t*	df	*t*	df
Multiple non-disabled sibs	.02	688	.47	677	− .39	679	− 2.17*	672
Multiple disabled sibs	− .17	689	.31	678	− .80	680	− 1.25	691
Sib is older/younger	1.48	664	− .37	653	− .22	655	− .82	666
Sib gender	− 1.69	148	.74	708	1.09	712	− 2.48*	725
IDDsib gender	− .41	684	− .89	673	− 1.18	556	− 1.84	686
Same/different gender	.25	691	.92	680	2.14*	621	.89	693
IMD bottom quintile	− 3.62***	99	2.24*	627	− 2.60**	632	− 2.97**	100
Sib degree education	.4.73***	724	− 3.82***	711	2.59**	211	3.02**	231
Sib lives with parents	− 4.00***	723	2.65**	710	− 1.17	715	.33	727
Sib lives with IDDsib	− 3.75***	722	2.13*	709	− 2.28*	713	− .92	726
IDDsib autism	− 2.01*	725	2.49*	712	− 1.72	716	− .23	729
IDDsib Down syndrome	4.19***	725	− 5.64***	461	3.23**	716	4.25***	554
IDDsib Cerebral Palsy	− 2.25*	725	3.44**	712	− 3.19**	716	− 2.81**	74
Sibling is a carer	− 3.25**	680	1.89	669	− 3.68***	671	− 3.59***	671

	Pearson’s r	*N*	Pearson’s r	*N*	Spearman’s r	*N*	Spearman’s r	*N*
Sib age	− .17***	727	.06	714	.03	718	.08*	731
IDDsib age	− .14***	692	.04	681	.03	683	.09*	694
Subjective poverty	.35***	708	− .36***	695	.44***	700	.34***	712
Independence IDDsib	− .16***	690	.17***	679	− .17***	681	− .08*	692
IDDsib *n* health conditions	.10*	665	− .15***	654	.16***	656	.13***	667

*p =  < .05, **p =  < .01, ***p =  < .001

Table 2 provides a correlation matrix of the four adult sibling outcome variables: mental distress, wellbeing, quality of life, and health as well as the three main predictor variables: carer status, subjective poverty, and level of independence of the brother or sister with IDD. The test variables were statistically significantly correlated with the four sibling outcome variables providing further support to include these variables in the final analysis models.

**Table 2:** Correlation matrix of dependent variables and main predictors

	1	2	3	4	5	6	7
1. Mental distress	–						
2. Wellbeing	− .72***	–					
3. Quality of life	.59***	− .64***	–				
4. Health	.49***	− .45***	.52***	–			
5. Sibling is a carer	.12**	− .07	.14***	.14***	–		
6. Subjective poverty	.35***	− .36***	.44***	.36***	.12**	–	
7. Independence of IDD sib	− .16***	.17***	− .17***	− .08*	− .19***	− .05	–

*p =  < .05, **p =  < .01, ***p =  < .001

#### Moderated Multiple Regression Analyses

Tables 3 and 4 present the findings from the moderated multiple regressions for each of the four sibling outcomes. All models overall accounted for a significant proportion of variance in the relevant outcome, with R^2^ in the range 20%–28%. Being a carer independently predicted sibling health (*β* = 0.18, *t*(569) = 2.46, *p* = 0.014), but not mental distress, wellbeing, or quality of life. Subjective poverty independently predicted all four sibling outcomes: mental distress (*β* = 1.10, *t*(568) = 7.45, *p* < 0.001), wellbeing (*β* = −0.86, *t*(557) = −7.98, *p* < 0.001), quality of life (*β* = 0.27, *t*(561) = 11.32, *p* < 0.001), and health (*β* = 0.20, *t*(569) = 7.51, *p* < 0.001). The level of independence in daily living (W-ADL) of the brother/sister with IDD independently predicted sibling mental distress (*β* = − 0.08, *t*(568) =  −3.44, *p* = 0.001), wellbeing (*β* = 0.06, *t*(557) = 3.34, *p* = 0.001), and quality of life (*β* = − 0.01, *t*(561) = − 3.377, *p* =  < 0.001), but not sibling health (*β* = 0.00, *t*(569) = 0.24, *p* = 0.809).

**Table 3:** Moderated multiple regressions for sibling mental distress and wellbeing

	Mental distress (K6)^a^						Wellbeing (SWEMWBS)^b^					
	*β*	SE	*t*	*p*	95% LLCI	95% ULCI	*β*	SE	*t*	*p*	95% LLCI	95% ULCI
Constant	10.37	1.60	6.47	< .001	7.22	13.52	21.28	1.16	18.28	< .001	18.99	23.57
Sibling is a carer	0.42	0.39	1.08	.281	− 0.35	1.19	− 0.06	0.29	− 0.22	.825	− 0.62	0.50
Subjective poverty	1.10	0.15	7.45	< .001	0.81	1.38	− 0.86	0.11	− 7.98	< .001	− 1.07	− 0.65
Carer × poverty	− 0.64	0.29	− 2.24	.026	− 1.20	− 0.08	0.55	0.21	2.62	.009	0.14	0.96
Independence of IDD sib	− 0.08	0.02	− 3.44	.001	− 0.13	− 0.04	0.06	0.02	3.34	.001	0.02	0.09
Carer × independence	0.01	0.05	0.28	.777	− 0.08	0.10	− 0.03	0.03	− 0.79	.432	− 0.09	0.04
Model covariates												
Sib age	− 0.06	0.02	− 3.54	< .001	− 0.09	− 0.03	0.00	0.01	0.07	.943	− 0.02	0.02
Sib has a non-disabled sib	− 0.45	0.40	− 1.13	.260	− 1.22	0.33	0.08	0.29	0.26	.795	− 0.49	0.64
Sib gender	0.97	0.54	1.79	.074	− 0.10	2.04	− 0.45	0.40	− 1.12	.263	− 1.23	0.34
Same/different gender	− 0.38	0.39	− 0.97	.332	− 1.14	0.39	− 0.11	0.28	− 0.41	.685	− 0.67	0.44
Neighborhood deprivation	0.90	0.59	1.53	.126	− 0.25	2.05	− 0.02	0.42	− 0.06	.954	− 0.85	0.80
Sib education	− 1.19	0.50	− 2.40	.017	− 2.16	− 0.21	1.03	0.36	2.86	.004	0.32	1.75
Sib lives with parents	− 0.39	0.62	− 0.63	.528	− 1.61	0.83	0.36	0.46	0.78	.434	− 0.54	1.25
Sib lives with IDD sib	0.68	0.64	1.07	.285	− 0.57	1.94	− 0.68	0.47	− 1.45	.147	− 1.60	0.24
IDD sib autism	− 0.21	0.43	− 0.50	.617	− 1.05	0.62	0.01	0.31	0.02	.982	− 0.61	0.62
IDD sib Down syndrome	− 0.96	0.45	− 2.11	.036	− 1.85	− 0.06	1.23	0.33	3.71	< .001	0.58	1.88
IDD sib cerebral palsy	− 0.08	0.71	− 0.12	.905	− 1.47	1.31	− 0.32	0.52	− 0.61	.541	− 1.35	0.71

Model summaries (all predictors to Y):

^a^*F*(16, 551) = 9.16, *p* < .001, *R*^*2*^ = .21, *N* = 568;

^b^ *F*(16, 540) = 9.53, *p* < .001, *R*^*2*^ = .22, *N* = 557

**Table 4:** Moderated multiple regressions for sibling quality of life and health

	Adult sibling quality of life^a^						Adult sibling health^b^					
	*β*	SE	*t*	*p*	95% LLCI	95% ULCI	*β*	SE	*t*	*p*	95% LLCI	95% ULCI
Constant	2.17	0.26	8.39	< .001	1.66	2.68	1.60	0.29	5.46	< .001	1.02	2.17
Sibling is a carer	0.05	0.06	0.74	.459	− 0.08	0.17	0.18	0.07	2.46	.014	0.04	0.32
Subjective poverty	0.27	0.02	11.32	< .001	0.22	0.32	0.20	0.03	7.51	< .001	0.15	0.25
Carer × poverty	− 0.09	0.05	− 1.83	.068	− 0.18	0.01	− 0.06	0.05	− 1.10	.272	− 0.16	0.05
Independence of IDD sib	− 0.01	0.00	− 3.77	< .001	− 0.02	− 0.01	0.00	0.00	0.24	.809	− 0.01	0.01
Carer × Independence	0.01	0.01	0.85	.394	− 0.01	0.02	0.01	0.01	1.38	.168	0.00	0.03
*Model covariates*												
Sib age	0.01	0.00	2.81	.005	0.00	0.01	0.01	0.00	3.05	.002	0.00	0.02
Sib has a non-disabled sib	− 0.06	0.06	− 0.97	.335	− 0.19	0.06	0.08	0.07	1.11	.266	− 0.06	0.22
Sib gender	− 0.09	0.09	− 1.05	.294	− 0.27	0.08	0.18	0.10	1.84	.066	− 0.01	0.38
Same/different gender	− 0.14	0.06	− 2.28	.023	− 0.27	− 0.02	− 0.06	0.07	− 0.86	.389	− 0.20	0.08
Neighborhood deprivation	0.01	0.09	0.14	.891	− 0.17	0.20	0.18	0.11	1.73	.084	− 0.02	0.39
Sib education	− 0.18	0.08	− 2.26	.024	− 0.34	− 0.02	− 0.16	0.09	− 1.82	.070	− 0.34	0.01
Sib lives with parents	− 0.09	0.10	− 0.86	.388	− 0.28	0.11	− 0.18	0.11	− 1.57	.117	− 0.40	0.04
Sib lives with IDD sib	0.24	0.10	2.36	.018	0.04	0.45	0.21	0.12	1.81	.072	− 0.02	0.44
IDD sib autism	0.05	0.07	0.78	.436	− 0.08	0.19	0.00	0.08	0.05	.962	− 0.15	0.16
IDD sib Down syndrome	− 0.06	0.07	− 0.82	.414	− 0.20	0.08	− 0.20	0.08	− 2.41	.016	− 0.36	-0.04
IDD sib Cerebral Palsy	0.12	0.12	1.06	.289	− 0.1	0.35	0.36	0.13	2.80	.005	0.11	0.62

Model summaries (all predictors to Y):

^a^*F*(16, 544) = 13.46, *p* < .001, *R*^*2*^ = .28, *N* = 561

^b^*F*(16, 552) = 8.78, *p* < .001, *R*^*2*^ = .20, *N* = 569

Subjective poverty was found to moderate the association between status as a carer and mental distress (*F*(1, 551) = 5.01, *p* = 0.026, change *R*^2^ = 0.01), wellbeing (*F*(1, 540) = 6.89, *p* = 0.009, change *R*^2^ = 0.01) but not quality of life (*F*(1, 544) = 3.34, *p* = 0.068, change *R*^2^ = 0.00) or health (*F*(1, 552) = 1.21, *p* = 0.272, change *R*^2^ = 0.00). These two models showed the same pattern, whereby when adult siblings reported *low* levels of subjective poverty, status as a carer was associated with higher mental distress (*t* = 2.49, *p* = 0.013) and lower wellbeing (*t* = − 2.23, *p* = 0.026). At average and high levels of subjective poverty, the association between carer status and outcomes was statistically non-significant. There was no evidence that the level of independence of the brother/sister with IDD moderated the association between status as a carer and sibling outcomes.

No covariate was a statistically significant predictor in all four models. Younger siblings reported higher mental distress (*β* = − 0.06, *t*(568) = − 3.54, *p* < 0.001), better quality of life (*β* = 0.01, *t*(561) = 2.81, *p* = 0.005) and better health (*β* = 0.01, *t*(569) = 3.05, *p* = 0.002). Siblings with degree-level qualifications reported lower levels of mental distress (*β* = − 1.19, *t*(568) = − 2.40, *p* = 0.017) than siblings without degree-level qualifications, higher wellbeing (*β* = 1.03, *t*(557) = 2.86, *p* = 0.004), and better quality of life (*β* = − 0.18, *t*(561) = –2.26, *p* = 0.024). Siblings who lived with their brother or sister with IDD reported worse quality of life than siblings that did not live with their brother or sister with IDD (*β* = 0.24, *t*(561) = 2.36, *p* = 0.018). Siblings who had a brother or sister with Down syndrome compared to other conditions reported lower mental distress (*β* = − 0.96, *t*(568) = − 2.11, *p* = 0.036), higher wellbeing (*β* = 1.23, *t*(557) = 3.71, *p* < 0.001), and better health (*β* = − 0.20, *t*(569) = − 2.41, *p* = 0.016). Siblings who had a brother or sister with Cerebral palsy compared to other conditions reported worse health (*β* = 0.36, *t*(569) = 2.80, *p* = 0.005).

### Research Question 2

Table 5 provides descriptive statistics and compares each of the four sibling outcomes—mental distress, wellbeing, quality of life, and health—with normative samples. Mean comparisons indicate that adult siblings had poorer scores on all four outcomes. For mental distress, adult siblings had higher mental distress scores (mean = 8.23, *SD* = 4.88, *N* = 727) compared to other adults (mean = 2.90, *SD* = 4.08, *N* = 24,761). The difference between the two groups had a large effect size (Cohen’s *d* = 1.19). Adult siblings also had worse wellbeing (mean = 21.30, *SD* = 3.50, *N* = 714) compared to other English adults (mean = 23.61, *SD* = 3.90, *N* = 7196). Therefore, the adult siblings of people with IDD had worse wellbeing than other adults. The effect size for this difference was of a moderate size (*d* = 0.62). Although for quality of life and health, siblings of people with IDD had statistically significantly worse outcomes compared to other adults, the effect sizes for these group differences were small. Table 5 also compares adult siblings who were carers for their brothers/sisters with IDD with adult siblings who were not carers across the four sibling outcomes: mental distress, wellbeing, quality of life, and health. For mental distress, quality of life, and health, sibling carers had statistically significantly worse outcomes compared to siblings that were not carers. However, the effect sizes for all four comparisons were small.

**Table 5:** Sibling outcomes compared with normative samples and group comparisons between being a caregiver or not with sibling outcomes

	Current sample mean (*SD*)	*t*	Normative mean (*SD*)	Cohen’s *d*	Carer mean (*SD*)	Not a carer mean (*SD*)	*t*	Cohen’s *d*
Mental distress	8.23 (4.88)	45.48***	2.90 (4.08)^a^	1.19	8.72 (4.90)	7.58 (4.78)	− 3.25**	.24
Wellbeing	21.30 (3.50)	162.46***	23.61 (3.90)^b^	.62	21.05 (3.52)	21.57 (3.54)	1.89	.15
Quality of Life	2.04 (.85)	64.47***	1.94 (.91)^c^	.11	2.15 (.84)	1.91 (.82)	− 3.68***	.29
Health	2.10 (.89)	64.05***	1.95 (.95)^d^	.16	2.19 (.91)	1.95 (.84)	− 3.59***	.27

*p =  < .05, **p =  < .01, ***p =  < .001;

Comparison from the following datasets:

^a^NCHS, ‘National Health Interview Survey, 2018’ (2020)

^b^Health Survey for England data, 2011 (Warwick Medical School, 2020)

^c^‘Survey of Carers in Households, 2009–2010’ (Information Centre for Health and Social Care, GfK NOP, 2011)

^d^‘Health Survey for England, 2018’ (2020)

Sibling sample for each outcome: Mental distress N = 727; Wellbeing N = 714; Quality of Life N = 718; Health N = 731

## Discussion

For our main analyses, there was evidence of a moderating effect of subjective poverty between carer status and adult sibling outcomes (mental distress and wellbeing). In each model, we found that where adult siblings had low levels of subjective poverty, carer status was associated with siblings’ mental distress, wellbeing, and quality of life. We expected to see that carer status would be associated with poorer sibling outcomes at *high* levels of subjective poverty because we hypothesized that there may be a ‘pile up’ (Double ABCX model) or cumulative stressors acting in concert. This suggests that for siblings who are financially managing or comfortable, sibling support should concentrate on those who also have a caring role for their brother or sister with IDD.

However, independent of other variables, sibling participants with high levels of subjective poverty were at a heightened risk of worse outcomes across all four models. This is perhaps indicative of the ways in which deprivation and poverty are risk factors for worse mental health outcomes more generally (Skapinakis et al., 2006). Therefore, it may be that the stresses related to poverty were so impactful in the lives of the sibling participants experiencing higher levels of poverty that the stresses of being a carer had less additional impact (i.e., that there was almost a ceiling effect of stress). As adult siblings experiencing poverty were at the highest risk of experiencing worse outcomes, whether or not they were a carer for their brother or sister with IDD, it is important for sibling support work to focus a significant amount of their time and resources on siblings who are experiencing poverty or socio-economic deprivation.

The independent associations of the level of independence of the brother or sister with IDD were statistically significant in the mental distress, wellbeing, and quality of life models, but not in the health model. Siblings that had brothers or sisters with IDD with higher levels of independence had slightly less mental distress, better wellbeing, and better quality of life. We expected that siblings having a brother or sister with IDD and higher levels of independence would have better outcomes, because the types of care and support that siblings may provide for their brothers and sisters with IDD may not be as extensive, and therefore less impactful on siblings’ outcomes. Siblings of people with less independence, and by implication, higher support needs, are therefore also candidates for further support.

Contrary to our initial hypothesis, no evidence was found that the level of independence of the brother/sister with IDD moderated the association between carer status and the sibling outcomes. We had hypothesized that siblings who were carers of a brother or sister with IDD who had lower levels of independence may be required to undertake more intensive levels of care, and that this may have more of an impact on their mental and physical health. Perhaps this is related to the social welfare available in the UK for disabled people with lower levels of independence. This may mean that the amount or the type of care that these siblings provided is of a different nature, or less intense, than we might have assumed. Unfortunately, we did not include questions about the supports and services the individuals with IDD were receiving and so cannot examine this hypothesis.

The following covariates were statistically significant in at least one model: age of sibling, sibling educational qualification level, sibling living with their brother or sister with IDD, the brother or sister with IDD having Down syndrome, or having Cerebral palsy. Interestingly, autism was not statistically significant in any of the four outcome models. Existing studies have explored the outcomes of siblings of people with Down syndrome in comparison to the siblings of people with autism (Hodapp & Urbano, 2007; Orsmond & Seltzer, 2007) and have found evidence of the Down syndrome advantage, whereby family members of people with Down syndrome fair better than family members of people with other conditions. Population-based data about child siblings has also found evidence of the Down syndrome advantage (Marquis et al., 2020). We have also found evidence of the Down syndrome ‘advantage’ in the models measuring siblings’ mental distress, wellbeing, and health, but not quality of life. This suggests that although we can conceptualize a Down syndrome ‘advantage’ for adult siblings, it would not be appropriate to conceptualize autism as a risk factor for worse sibling outcomes, supporting a need to resist a ‘negative narrative’ (Hastings, 2016) of the effect having a family member with IDD may have on an individual.

Overall (Table 5), adult siblings had worse scores in comparison to normative samples across all four measures of mental distress, wellbeing, quality of life, and health, although the effect sizes for quality of life and health were small. Like Rai et al., (2018) adult siblings reported worse mental distress than other adults. For health, Hodapp et al., (2010) found that most adult siblings reported good health. We also found that most siblings reported good health, but it was slightly worse than other adults, although with a small effect size. These findings provide some evidence that adult siblings, and adult sibling carers in particular, are in need of supports, particularly related to their mental distress and wellbeing, where the effect sizes were large and medium respectively.

We also explored the mental distress, wellbeing, quality of life, and health outcomes of adult siblings who were and were not carers (Table 5). Siblings with carer status had slightly worse outcomes than siblings without carer status for mental distress, quality of life, and health, with small effect sizes across these outcomes. However, once we controlled for other covariates and moderating variables using moderated multiple regression (Tables 3 and 4), carer status, independent of all other variables, was only a statistically significant predictor for one outcome: sibling health.

### Limitations

Most of the limitations for this study stem from the use of a survey study design and convenience sampling. Data for this study were from cross-sectional data only. Longitudinal data would be required to understand these associations further. All data were from sibling self-report data. We have no diagnostic information from relevant practitioners about the sibling (e.g., mental health diagnoses) or their brother or sister with IDD (e.g., to confirm they have an autism diagnosis). Our sample is also not likely to be representative of the UK population. We disproportionately recruited white, female, and well-educated participants and therefore we are unlikely to be able to generalize these findings to all UK siblings. We recruited through a national sibling charity, and we asked other disability support organizations to share the survey advertisement as well. Therefore, our participants were likely accessing or receiving communication from support organizations. People that take part in IDD sibling research are also more likely to self-identify as ‘siblings’, and perhaps feel more affected by their experiences as siblings than other siblings. The non-representativeness of our sample also limits our ability to compare our adult sibling sample outcomes to the normative sample outcomes presented in Table 5. Caution should be applied to comparing these groups, with the worse outcomes found for adult siblings in comparison to the normative samples potentially being an over- or an under-estimate of true differences.

In terms of the sample of adult siblings who started the survey, there was also a significant sub-group who did not progress after questions asking about family income. The potential effect of this is unknown in the current study. However, all of the cautions made about the overall likely non-representative nature of the sample are reinforced by this data missingness pattern.

A problem with caregiving research, and sibling caregiving research specifically, is that caregiving is not always defined for participants (Lee & Burke, 2018) and where there is a definition of caregiving provided, it varies across studies (Lee & Burke, 2018). Although our study did provide a UK national definition of informal caregiving to guide participants, respondents were only able to select ‘Yes’ or ‘No’ in response to whether they felt the definition applied to them. Our binary definition is arguably ‘overly simplistic’ (Lee et al., 2018: 126). Other studies have probed the concept of caregiving further, exploring the concept as more of a continuum rather than as a binary construct (Lee et al., 2018).

Only about 20%–28% of the variance was explained by the moderated multiple regression analyses and our effect sizes were small. This indicates that there are other factors explaining siblings’ outcomes, and these may not be related to sibling status or carer status.

### Research Implications

A conceptual issue with this study is that by exploring sibling outcomes and finding that siblings have worse outcomes than other adults, we are implicated in advancing a ‘negative narrative’ (Hastings, 2016) about the effect of having a brother or sister with IDD. However, understanding more clearly the factors associated with the outcomes of a group that may potentially need further support, has importance and value. This is particularly so for siblings with carer status, who are relied on to support and care for their brothers and sisters with IDD. Further research is necessary to understand this finding further and consider why caring was associated with poorer outcomes for siblings with low levels of subjective poverty. Future research may consider including in analyses factors related to how siblings perceive the way that caring impacts their lives (i.e., carer ‘burden’ measures).

### Practical and Clinical Implications

The current data suggest that some siblings may be in need of supports and interventions, particularly related to contextual factors such as for those siblings experiencing socio-economic hardships. Siblings that have a brother or sister with IDD and *lower* levels of independence may also require more support. Our data also suggest that siblings that have low levels of subjective poverty but that are carers may benefit from specific supports as well. The mental distress and wellbeing outcomes had large and medium effect sizes when compared with other adults using national data, whereas the effect sizes in these comparisons were small for quality of life and health. Therefore, any interventions and supports might focus particularly on psychological outcomes related to mental distress and mental wellbeing.

## References

[CR1] Arnold CK, Heller T, Kramer J (2012). Support needs of siblings of people with developmental disabilities. Intellectual and Developmental Disabilities.

[CR2] Australian Institute of Family Studies (2011). *The longitudinal study of Australian children annual statistical report 2010*. Retrieved October 29, 2020, from https://growingupinaustralia.gov.au/sites/default/files/asr2010.pdf

[CR3] Burke MM, Taylor JL, Urbano R, Hodapp RM (2012). Predictors of future caregiving by adult siblings of individuals with intellectual and developmental disabilities. American Journal on Intellectual and Developmental Disabilities.

[CR4] Coppus AMW (2013). People with intellectual disability: What do we know about adulthood and life expectancy?. Developmental Disabilities Research Reviews.

[CR5] Cox MJ, Paley B (1997). Families as systems. Annual Review of Psychology.

[CR6] Coyle CE, Kramer J, Mutchler JE (2014). Aging together: Sibling carers of adults with intellectual and developmental disabilities. Journal of Policy and Practice in Intellectual Disabilities.

[CR7] Egan J, Walsh PN (2001). Sources of stress among adult siblings of Irish people with intellectual disability. The Irish Journal of Psychology.

[CR8] Galobardes B, Lynch J, Smith GD (2007). Measuring socioeconomic position in health research. British Medical Bulletin.

[CR9] Gill, B. (2015). *The English indices of deprivation 2015 [statistical release].* Department for communities and local government. Retrieved January 23, 2019, from https://www.gov.uk/government/statistics/english-indices-of-deprivation-2015

[CR10] Hastings RP (2016). Do children with intellectual and developmental disabilities have a negative impact on other family members? The case for rejecting a negative narrative. International Review of Research in Developmental Disabilities.

[CR11] Hayden NK, Hastings RP, Totsika V, Langley E (2019). A population-based study of the behavioral and emotional adjustment of older siblings of children with and without intellectual disability. Journal of Abnormal Child Psychology.

[CR12] Hayes AF (2018). Introduction to mediation, moderation, and conditional process analysis: A regression-based approach.

[CR13] Heller T, Arnold CK (2010). Siblings of adults with developmental disabilities: Psychosocial outcomes, relationships, and future planning. Journal of Policy and Practice in Intellectual Disabilities.

[CR14] Heller T, Kramer J (2009). Involvement of adult siblings of persons with developmental disabilities in future planning. Intellectual and Developmental Disabilities.

[CR15] Hodapp RM, Urbano RC (2007). Adult siblings of individuals with Down syndrome versus with autism: Findings from a large-scale US survey. Journal of Intellectual Disability Research.

[CR16] Hodapp RM, Urbano RC, Burke MM (2010). Adult female and male siblings of persons with disabilities: Findings From a national survey. Intellectual and Developmental Disabilities.

[CR17] Howlin P, Moss P, Savage S, Bolton P, Rutter M (2015). Outcomes in adult life among siblings of individuals with autism. Journal of Autism and Developmental Disorders.

[CR18] Information Centre for Health and Social Care, GfK NOP. (2011). ‘*Survey of carers in households, 2009-2010’*. [data collection]. UK Data Service. SN: 6768, http://doi.org/10.5255/UKDA-SN-6768-1

[CR19] Kessler RC, Barker PR, Colpe LJ, Epstein JF, Gfroerer JC, Hiripi E, Howes MJ, Normand ST, Manderscheid RW, Walters EE, Zaslavsky AM (2003). Screening for serious mental illness in the general population. Archives of General Psychiatry..

[CR20] Lee C, Burke MM (2018). Caregiving roles of siblings of adults with intellectual and developmental disabilities: A systematic review. Journal of Policy and Practice in Intellectual Disabilities.

[CR21] Lee C, Burke MM (2020). Future planning among families of individuals with intellectual and developmental disabilities: A systematic review. Journal of Policy and Practice in Intellectual Disabilities.

[CR22] Lee C, Burke MM, Arnold CK (2019). Examining the relation between disability severity among older adults with disabilities and sibling caregiving. Research and Practice for Persons with Severe Disabilities.

[CR23] Lee C, Burke MM, Arnold CK, Owen A (2018). Perceptions of non-caregiving roles among siblings of adults with intellectual and developmental disabilities. Research and Practice in Intellectual and Developmental Disabilities.

[CR24] Lee C, Burke MM, Arnold CK, Owen A (2020). Compound sibling careers of individuals with intellectual and developmental disabilities. Journal of Applied Research in Intellectual Disabilities.

[CR25] Maenner MJ, Smith LE, Hong J, Makuch R, Greenberg JS, Mailick MR (2013). Evaluation of an activities of daily living scale for adolescents and adults with developmental disabilities. Disability and Health Journal.

[CR26] Marquis S, McGrail K, Hayes MV (2020). Using administrative data to examine variables affecting the mental health of siblings of children who have a developmental disability. Research in Developmental Disabilities.

[CR27] McCubbin HI, Patterson JM (1983). The family stress process: The double ABCX model of adjustment and adaptation. Marriage & Family Review.

[CR28] MCS (2017). *Welcome to the Millennium Cohort Study.* Retrieved January 23, 2019, from https://cls.ucl.ac.uk/cls-studies/millennium-cohort-study/

[CR29] National Center for Health Statistics (2020). *NCHS, National Health Interview Survey, 2018.* NHIS Data, Questionnaires and related documentation. Retrieved June 29, 2020, from https://www.cdc.gov/nchs/nhis/data-questionnaires-documentation.htm

[CR30] National Centre for Social Research (NatCen), University College London, Department of Epidemiology and Public Health. (2020). ‘Health Survey for England, 2018’. [data collection]. UK Data Service. SN: 8649, 10.5255/UKDA-SN-8649-1

[CR31] NHS England (2020). *Who is considered a carer?* nhs.uk. Retrieved June 24, 2020, from https://www.england.nhs.uk/commissioning/comm-carers/carers/#:~:text=A%20carer%20is%20anyone%2C%20including,care%20they%20give%20is%20unpaid

[CR32] O’Neill LP, Murray LE (2016). Anxiety and depression symptomatology in adult siblings of individuals with different developmental disability diagnoses. Research in Developmental Disabilities.

[CR33] Orsmond GI, Seltzer MM (2007). Siblings of individuals with autism or Down syndrome: Effects on adult lives. Journal of Intellectual Disability Research.

[CR34] Prino LE, Scigala D, Fabris MA, Longobardi C (2019). The moderating role of gender in siblings of adults with intellectual disabilities. Interpersona: An International Journal on Personal Relationships.

[CR35] Rai D, Heuvelman H, Dalman C, Culpin I, Lundberg M, Carpenter P, Magnusson C (2018). Association between autism spectrum disorders with or without intellectual disability and depression in young adulthood. JAMA Network Open.

[CR36] Rimmerman A, Raif R (2001). Involvement with and role perception toward an adult sibling with and without mental retardation. The Journal of Rehabilitation.

[CR37] Seltzer GB, Begun A, Seltzer MM, Krauss MW (1991). Adults with mental retardation and their aging mothers: Impacts of siblings. Family Relations.

[CR38] Seltzer MM, Greenberg JS, Hong J, Smith LE, Almeida DM, Coe C, Stawski RS (2010). Maternal cortisol levels and behavior problems in adolescents and adults with ASD. Journal of Autism and Developmental Disorders.

[CR39] Skapinakis P, Weich S, Lewis G, Singleton N, Araya R (2006). Socio-economic position and common mental disorders: Longitudinal study in the general population in the UK. The British Journal of Psychiatry.

[CR40] Sonik RA, Parish SL, Rosenthal ES (2016). Sibling caregivers of people with intellectual and developmental disabilities: Sociodemographic characteristics and material hardship prevalence. Intellectual and Developmental Disabilities.

[CR41] Stewart-Brown S, Tennant A, Tennant R, Platt S, Parkinson J, Weich S (2009). Internal construct validity of the Warwick-Edinburgh mental well-being scale (WEMWBS): A Rasch analysis using data from the Scottish health education population survey. Health and Quality of Life Outcomes.

[CR42] Taggart L, Truesdale-Kennedy M, Ryan A, McConkey R (2012). Examining the support needs of ageing family careers in developing future plans for a relative with an intellectual disability. Journal of Intellectual Disabilities.

[CR43] Taylor JL, Greenberg JS, Seltzer MM, Floyd FJ (2008). Siblings of adults with mild intellectual deficits or mental illness: Differential life course outcomes. Journal of Family Psychology.

[CR44] Tomeny TS, Ellis BM, Rankin JA, Barry TD (2017). Sibling relationship quality and psychosocial outcomes among adult siblings of individuals with autism spectrum disorder and individuals with intellectual disability without autism. Research in Developmental Disabilities.

[CR45] Totsika V, Hastings RP, Vagenas D (2016). Informal caregivers of people with an intellectual disability in England: Health, quality of life and impact of caring. Health and Social Care in the Community.

[CR46] Warwick Medical School (2020). *SWEMWBS population norms in Health Survey for England data 2011*. University of Warwick*.* Retrieved October 29, 2020, from https://warwick.ac.uk/fac/sci/med/research/platform/wemwbs/using/howto/wemwbs_population_norms_in_health_survey_for_england_data_2011.pdf

